# Proportional myoelectric control of a virtual bionic arm in participants with hemiparesis, muscle spasticity, and impaired range of motion

**DOI:** 10.1186/s12984-024-01529-0

**Published:** 2024-12-21

**Authors:** Caleb J. Thomson, Fredi R. Mino, Danielle R. Lopez, Patrick P. Maitre, Steven R. Edgley, Jacob A. George

**Affiliations:** 1 Department of Biomedical Engineering, University of Utah, Salt Lake City, UT USA; 2 Department of Electrical and Computer Engineering, University of Utah, Salt Lake City, UT USA; 3 Interdepartmental Neuroscience Program, University of Utah, Salt Lake City, UT USA; 4 Department of Physical Medicine and Rehabilitation, University of Utah, Salt Lake City, UT USA; 5 Department of Mechanical Engineering, University of Utah, Salt Lake City, UT USA

**Keywords:** Myoelectric, EMG, Hemiparesis, Stroke, Motor control, Exoskeleton, Powered orthosis

## Abstract

**Background:**

This research aims to improve the control of assistive devices for individuals with hemiparesis after stroke by providing intuitive and proportional motor control. Stroke is the leading cause of disability in the United States, with 80% of stroke-related disability coming in the form of hemiparesis, presented as weakness or paresis on half of the body. Current assistive exoskeletonscontrolled via electromyography do not allow for fine force regulation. Current control strategies provide only binary, all-or-nothing control based on a linear threshold of muscle activity.

**Methods:**

In this study, we demonstrate the ability of participants with hemiparesis to finely regulate their muscle activity to proportionally control the position of a virtual bionic arm. Ten stroke survivors and ten healthy, aged-matched controls completed a target-touching task with the virtual bionic arm. We compared the signal-to-noise ratio (SNR) of the recorded electromyography (EMG) signals used to train the control algorithms and the task performance using root mean square error, percent time in target, and maximum hold time within the target window. Additionally, we looked at the correlation between EMG SNR, task performance, and clinical spasticity scores.

**Results:**

All stroke survivors were able to achieve proportional EMG control despite limited or no physical movement (i.e., modified Ashworth scale of 3). EMG SNR was significantly lower for the paretic arm than the contralateral nonparetic arm and healthy control arms, but proportional EMG control was similar across conditions for hand grasp. In contrast, proportional EMG control for hand extension was significantly worse for paretic arms than healthy control arms. The participants’ age, time since their stroke, clinical spasticity rate, and history of botulinum toxin injections had no impact on proportional EMG control.

**Conclusions:**

It is possible to provide proportional EMG control of assistive devices from a stroke survivor’s paretic arm. Importantly, information regulating fine force output is still present in muscle activity, even in extreme cases of spasticity where there is no visible movement. Future work should incorporate proportional EMG control into upper-limb exoskeletons to enhance the dexterity of stroke survivors.

**Supplementary Information:**

The online version contains supplementary material available at 10.1186/s12984-024-01529-0.

## Introduction

Stroke is the leading cause of disability in the United States, with more than 795,000 people suffering a stroke each year [1]. 80% of stroke-related motor deficits are in the form of upper-limb hemiparesis [1, 2]. Hemiparesis presents as a one-sided weakness or paralysis and is caused by damage to the central nervous system from the stroke. This damage to the central nervous system interrupts descending motor control, dissociates motor responses and sensory inputs, and can lead to hyperexcitability of the muscles, causing spasticity [3, 4].

After a stroke, residual muscle activity in the hemiparetic arm can be recorded using surface electromyography (EMG), even in patients with no detectable muscle activity as measured by traditional clinical assessments [5]. Even in the chronic phase, muscle activity persists and can be improved over time [6, 7]. However, the ability to modulate muscle activity is diminished in chronic stroke patients [8], which can lead to abnormal muscle activations and task difficulty [9]. Motor deficits have also been found in the nonparetic arm after a stroke [10].

Ultimately, hemiparesis makes it difficult to complete activities of daily living, reducing the quality of life and autonomy [11]. Assistive devices, like powered orthoses [12, 13] and functional electrical stimulation (FES) [14], have been used to restore hand function to stroke patients with hemiparesis, thereby restoring independence and increasing quality of life [13, 15, 16]. Because muscle activity still persists in hemiparetic stroke patients [5], EMG can serve as an intuitive control signal for FES [17, 18] and powered orthoses [13, 19]. However, one challenge when using paretic EMG for control is the presence of involuntary EMG increases when the individual moves another part of their arm [20]; this has been shown to decrease the accuracy of EMG-based control algorithms [21].

Due to the complexities of EMG and the abnormalities of paretic EMG [8, 20, 22–25], current EMG control algorithms most often employ a binary, “all-or-nothing” approach that simply detects if the muscle is active or inactive. When this binary control is used to control the position of a hand exoskeleton, individuals are limited to maximally closing or maximally opening their hand. Because there is a fixed force output from the exoskeleton, binary control makes it difficult, if not impossible, to perform fine motor actions. Variable force output is critical in tasks like manipulating fragile objects [26], preventing slips [27], and grasping under uncertain conditions [28].

Pattern recognition has been used to extract more precise control from EMG activity for research applications with exoskeletons [29–31] and commercial applications with prostheses [32–34]. However, pattern recognition systems still provide only discrete class predictions and do not inherently provide proportional position control or fine force regulation. Proportional control of upper-limb exoskeletons has been shown with proportional force [35–37], torque [38, 39], velocity [40, 41], and position control [41, 42]. The proportional control demonstrated in these studies mainly focuses on the arm from the wrist up to the shoulder; those that do look at control of the hand use force [37], torque [39], and velocity control [40], and not position control. The joint from the wrist to the shoulder collectively supports gross motor function (i.e., positioning of the hand in space) and leverages large, anatomically distinct muscles for control (e.g., the biceps and triceps). In contrast, fine motor control of the hand involves the coordination of multiple small muscles densely packed in the forearm. In the adjacent field of upper-limb prosthetic control, proportional position control is common [42–47] and has been shown to increase performance relative to velocity control for a prosthetic hand [48]. Proportional position control is also more closely aligned with the natural encoding for hand control, which is in terms of joint position [49–51].

A key challenge in realizing proportional position control for upper-limb exoskeletons is that the primary patient population, stroke patients, often has severe muscle spasticity [52, 53]. Muscle spasticity often manifests as lower EMG SNR [54], excessive co-contractions [55, 56], and delayed muscle activation/relaxation [22–25, 56]. Due to these signal challenges, proportional position control of exoskeletons has often been performed using EMG from the nonparetic, contralateral limb [31, 57, 58] rather than the affected paretic limb, which limits the ability to perform bilateral tasks. Others have only tested exoskeleton control with healthy participants rather than the target patient population of stroke survivors [30, 59].

Using high-density EMG in conjunction with machine learning can be a solution to obtain more robust and dexterous control from paretic EMG. High-density EMG gathers data from most, if not all, the muscles, and avoids the need to meticulously identify isolated EMG from desired muscles. Machine learning is then used to identify and exploit even the smallest differences among the ensemble of muscle activity when attempting different movements. Indeed, high-density EMG has already been used to classify hand gestures with high accuracy with paretic EMG from stroke survivors [29, 60]. Building on these works, here we propose high-density EMG in conjunction with machine learning to provide proportional control of the hand. To do this, we leverage a modified Kalman filter, which has been demonstrated to provide robust proportional position control with healthy individuals and with upper-limb amputees [46].

In this study, we specifically investigated the ability to extract proportional position control from the extrinsic hand muscles of stroke survivors with hemiparesis. We show that all participants were able to achieve proportional EMG control, regardless of their age, time since their stroke, clinical spasticity rate, and history of botulinum toxin injections. We also show that EMG signal-to-noise ratio and proportional control are better for hand grasp than hand extension, consistent with the neurophysiology of post-stroke spasticity [61]. These results can help guide the implementation and patient inclusion criteria for future assistive hand exoskeletons with proportional EMG control.

## Methods

### Participant information

Ten stroke survivors with hemiparesis were recruited for this study (Table 1). Additionally, ten healthy individuals with no neuromuscular impairments were recruited to serve as approximately age-matched controls (i.e., the ages were not matched exactly but the average age of both groups was similar) (Table 3). Informed consent and experimental protocols were carried out in accordance with the University of Utah Institutional Review Board and the Declaration of Helsinki guidelines. Table 1 lists the demographics of the stroke survivors, including age, sex, Modified Ashworth Scale score, type and location of stroke, and years post-stroke. Table 2 has botulinum injection information for the four participants who received injections. The age, sex, and handedness of the control group are found in Table 3.

**Table 1:** Stroke participant demographics

Participant	Age	Sex	MAS	Stroke type and location	Years since stroke
1	44	M	3	Unknown right	4.13
2	52	F	2	Ischemic Left MCA	1.41
3	24	F	1	Hemorrhagic right frontal lobe	5.40
4	56	F	3	Perioperative stroke	12.20
5	45	M	2.5	Ischemic Right MCA	1.21
6	32	M	1	Pediatric Hemorrhagic	30.66
7	57	M	1	Ischemic right MCA	0.15
8	47	F	3	Ischemic Right ICA	7.09
9	22	F	2	In utero Hemorrhagic	22.51
10	27	F	3	Abscess caused stroke left	3.20
Average	40.6 ± 13.28	60% F	2.15 ± 0.88	40% Ischemic, 40% hemorrhagic, 20% unknown	8.80 ± 10.18

**Table 2:** Botulinum toxin injection information for participants who received injections

Participant	Injection frequency	Days since injection at time of study	Muscles injected
5	3 months	52	Left FCR, Left FCU, Left FDS, Left, FDP
6	6 months	37	Left FDS
8	3 months	56	Left FDS, Left FDP, Left DI
10	3 months	100	Right Deltoid, Right FDS, Right FDP

Muscle abbreviations: FCR- Flexor carpi radialis, FCU- Flexor carpi ulnaris, FDS- Flexor digitorum superficialis, FDP – Flexor digitorum profundus, DI - Dorsal interossei

**Table 3:** Healthy control demographics

Control participant	Age	Sex	Handedness
1	74	F	L
2	32	M	R
3	27	M	L
4	45	F	R
5	64	M	R
6	35	F	R
7	54	F	R
8	23	F	R
9	26	F	L
10	28	F	L
Average	40.8 ± 17.74	60% F	60% R

### EMG sleeve and signal acquisition

Surface EMG from the participants was collected using custom EMG sleeves [62]. The EMG sleeve had 34 electrodes that make physical contact with the skin to record an electrical voltage. One electrode served as a permanent ground, and another served as a permanent reference. The remaining 32 electrodes produced a single-ended channel of EMG each using the permanent ground and reference. EMG was sampled at 1 kHz and filtered using the Summit Neural Interface Processor (Ripple Neuro, LLC, Salt Lake City, UT, USA). The recorded EMG was band-pass filtered with cutoff frequencies of 15 Hz (sixth-order high-pass Butterworth filter) and 375 Hz (second-order, low-pass Butterworth filter). Notch filters were applied at 60, 120, and 180 Hz to remove 60 Hz power-line interference and its second and third harmonics. Differential EMG signals were then calculated for all possible pairs of the 32 single-ended EMG channels, resulting in 496 (32 choose 2) differential recordings [46]. The mean absolute value (MAV) over a sliding 300-ms window was calculated at 30 Hz for all the single-ended channels and differential pairs. The resulting EMG feature set consisted of the 300-ms smoothed MAV on 528 EMG channels (32 channels from the 32 electrodes, and 496 channels from the differential pairs), calculated at 30 Hz, as has been done in prior work with upper-limb amputees [63].

### Training datasets

**Fig. 1:**
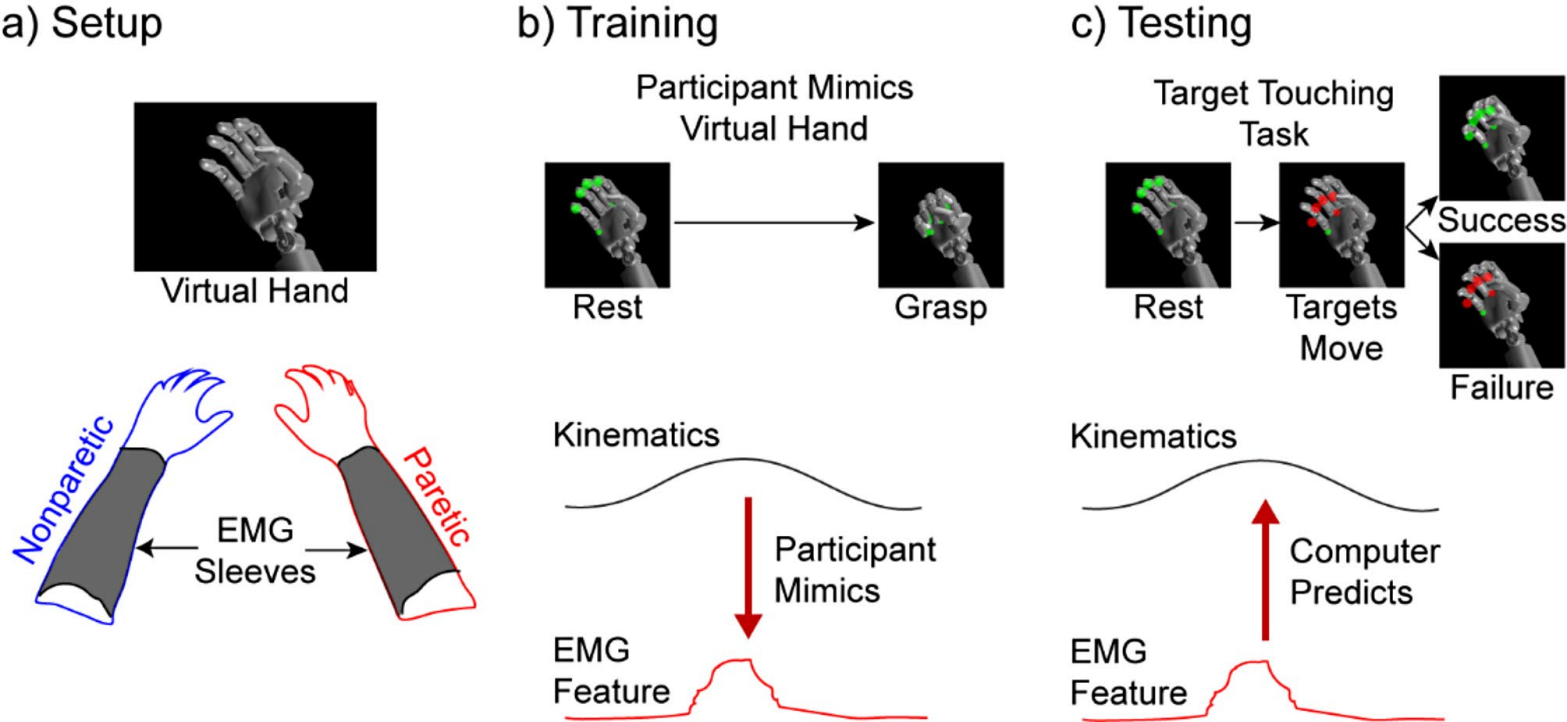
Experimental overview. (**A**) Participants who had hemiparesis due to a stroke completed the task with both their paretic and nonparetic arms. High-density EMG was recorded from the extrinsic hand muscles in the forearm using a custom EMG sleeve [62]. (**B**) The participants attempted to mimic preprogrammed kinematics of a bionic hand displayed on a computer screen during the training phase. EMG was recorded in synchrony, and a modified Kalman filter was trained to regress proportional position control from the ensemble of EMG activity [46]. (**C**) During the testing phase, the participants were given control of the bionic hand to complete a virtual target-touching task in real-time. The participants received visual cues for when each trial began and ended with circles indicating the targets would move from rest to the target location. Additionally, the color of the circles provided feedback to the participants if they were within the target window; red meant they were outside the target, and green meant they were inside the target window

The participants were instructed to mimic the programmed movements of a virtual bionic hand to correlate EMG activity to intended hand movements (Fig. 1). As the participants attempted to mimic the virtual hand with their paretic hand, we recorded, in synchrony, the kinematics (joint positions) of the virtual hand and EMG activity. The participants with hemiparesis completed two training sessions with each arm (four total), and the healthy controls completed two training sessions with their right arm only. Each training session consisted of grasping or extending all the digits of the hand ten times. Each movement was 4.4 s in duration, consisting of 0.7 s of grasp/extension away from the resting hand position, a 3-s hold-time at the maximum distance away from the resting hand position, and a 0.7-second relaxation returning to the resting hand position as described in [46]. The participants were given 2 s of rest between movements for the nonparetic arm and healthy arm, and 3 s of rest between movements for the paretic arm.

### Control algorithm

We used a Kalman Filter (KF) defined in prior work with upper-limb amputees [45, 46, 63–68] to estimate and predict motor intent from the continuous EMG signals. Ad-hoc modifications to this KF have been described in prior work [46], leading to the modified KF (MKF) used in this study. Briefly, the output is modified by a threshold such that the modified output will remain at zero until the absolute value of the nonmodified output is greater than the threshold. As in prior publications [63, 64], we used a default threshold value of 0.2. The MKF has been used previously for myoelectric control for upper limb prostheses [46, 63, 64, 69–71], and the detailed mathematical justification, construction, and parameters of the KF have been outlined in [65]. The baseline EMG MAV was subtracted from the EMG features before training and testing the KF. We assumed that the EMG features were normally distributed and relied on the KF covariance matrix to inherently address differences among them. The EMG feature set of 528 channels was reduced to 48 channels using a stepwise Gram-Schmidt channel-selection algorithm [72, 73]. At a high level, the Gram-Schmidt algorithm is an orthonormalization channel selection process that first maximizes correlation and then adds additional channels that contribute the most unique information. Briefly, during each iteration, estimates of each movement residual are made by projecting each feature residual onto the movement residual, and features are selected by finding the feature residuals that improve the RMSE between the movement residual and its estimate the most. The selected feature is added to a list and the process is iterated until a prescribed number of features is selected, in this case 48 (largest number of channels supported by the system for real-time inference). A single MKF was used to predict the position of the virtual hand. We limited outputs of the MKF between − 1 and 1, where − 1 corresponded to maximum extension, + 1 corresponded to maximum grasp, and 0 corresponded to when the hand was at rest [45]. We used 100% of the training data to train each MKF.

### Signal-to-noise ratio analysis

EMG signal-to-noise ratio (SNR) was calculated as the MAV of the EMG signal during movement divided by the MAV of the EMG signal during rest. EMG SNR was calculated for the 32 single-ended channels (e.g., one SNR value per electrode from each sleeve). EMG SNR was calculated separately for grasping (closing the hand) and extension (opening the hand). The median SNR from the 32 single-ended channels was used from each participant in the analysis. Additionally, further analysis was completed to explore the SNR for subsets of electrodes that would most likely be placed over a flexor or extensor muscle. To do this, the five highest correlated electrodes were selected using Pearson’s correlation coefficient for each movement, and the median SNR was reported for each participant. Similarly, the five channels with the highest SNR were also selected, and the median SNR was reported for each participant.

SNR was also calculated for the MKF and simple linear regression using one channel of EMG input. The linear regressor followed the format of $\:y=m*x+b$ where y was the kinematic position and x was the value of the single EMG channel. The slope (m) was calculated by dividing the covariance of x and y by the variance of x. The intercept was calculated by subtracting the slope times the average of x from the average of y. For both algorithms, 50% of the data was used to train and 50% was used for testing. The same training and testing trials were used for both algorithms. The EMG channel selected for the linear regressor was the channel with the highest Pearson’s correlation coefficient with the kinematic position.

### Real-time target touching task

We used a virtual target-touching task (TTT) to quantify user and algorithm performance. This task involves controlling a virtual bionic arm (MSMS; John Hopkins Applied Physics Lab, Baltimore, MD, USA), where the participant is provided real-time visual feedback. In this task, the participant actively controlled the virtual hand and attempted to move it to a target location and keep it there. Target locations were at 50% of the maximum grasp/extension possible to evaluate proportional control for the selected DOF. The participant was instructed to hold the hand in the target position for the trial duration. Visual feedback was provided to the participant to confirm that the hand was within ± 15% of the target location. Each test trial lasted 5 s, with a 2-s wait time between trials (10-s wait time for the paretic limb). A total of 20 trials were collected for both grasp and extension in groups of 10 trials to avoid participant fatigue. The participants completed the TTT for grasp and extension separately, and each participant was assigned pseudo randomly to begin with either grasp or extension.

We compared the performance of the paretic, nonparetic, and healthy controls using three metrics: (1) root mean square error (RMSE), (2) percent time in the target 15%-error window, and (3) the mean longest continuous-hold duration (i.e., hold duration) within the desired 15%-error window around the target location [64]. RMSE captures the ability to finely control the virtual hand. The percent time in target determines the overall performance on the task, and the mean longest continuous hold duration extrapolates performance to a more functional metric (such as the ability to hold an object without dropping it).

To calculate RMSE devoid of the participants’ reaction time, we delayed the recorded kinematics by a lag determined by cross-correlating the kinematic predictions and the target location signals. This alignment was applied across all experimental conditions for a given session so that no bias would affect one experimental condition more than another [46]. Additionally, the RMSE was calculated from the 15%-error window, such that the RMSE was zero if the hand was anywhere within the 15%-error window (consistent with the visual feedback the participants received) [74].

### Statistical analysis

All statistical analyses were completed using the Statistics and Machine Learning Toolbox in MATLAB 2021b (MathWorks, Natick, MA, USA).

The collapsed SNR data (i.e., *N* = 10) were determined to be nonparametric through the Anderson-Darling test (*p* < 0.05); therefore, nonparametric statistical tests were used. A one-way nonparametric analysis of variance (ANOVA; Kruskal-Wallis) was used to compare the EMG SNR for both grasping and extension of the hand for the three groups (paretic, nonparetic, healthy). If any significance was found, subsequent pairwise comparisons (Wilcoxon rank-sum tests) were made using Tukey’s honestly significant difference criterion correction for multiple comparisons. Additionally, the paretic SNR was split *post hoc* based on whether the participant received botulinum toxin injections (*N* = 4 with and *N* = 6 without). These data were also determined to be nonparametric, and nonparametric statistical tests were used. A Wilcoxon rank-sum test was used to compare the EMG SNR between participants receiving botulinum toxin injections and the SNR of participants who were not.

The collapsed TTT results for each outcome metric (i.e., *N* = 10) were determined to be parametric through the Anderson-Darling test (*p* > 0.05), so parametric statistical analyses were performed. A two-way analysis of variance (ANOVA) was used to compare task performance for the three groups (healthy controls, nonparetic, and paretic) and two movements (grasp and extension). The two factors were EMG source (healthy, nonparetic, and paretic) and movement type. If any significance was found, subsequent pairwise comparisons (two-sample *t*-test). Additionally, the variance was compared using population F-tests. As with the SNR data, the paretic TTT data was split *post hoc* by whether the participant received botulinum toxin injections (*N* = 4 with and *N* = 6 without). These data were also determined to be parametric, and parametric statistical tests were used. A two-sample *t*-test was used to compare the task performance between participants who had and had not received botulinum toxin injections.

The Pearson correlation coefficient was calculated between paretic and nonparetic performance metrics and demographic metrics of Modified Ashworth Scale score, age, time post-stroke, and sex.

## Results

### Paretic EMG SNR is significantly worse for hand extension, but not hand grasp

**Fig. 2:**
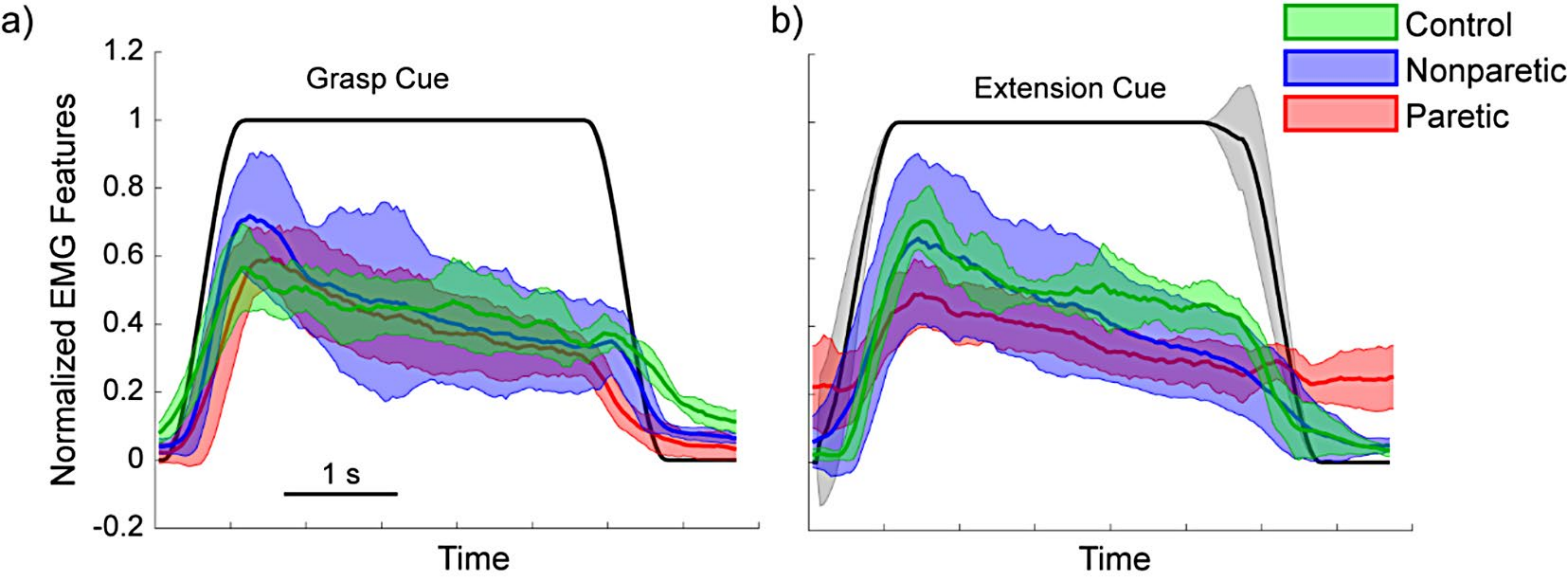
Normalized EMG activity from the extrinsic hand muscles during instructed hand grasp (**a**) and hand extension (**b**). Data show the EMG feature (300-ms smoothed mean absolute value) from one healthy control (green) and one stroke participant’s nonparetic (blue) and paretic (red) arms. Data show the mean and standard deviation from the 32 surface electrodes

We first compared the SNR of the EMG from healthy participants and the paretic and nonparetic arms of stroke participants performing hand grasps and extensions. Detectable EMG was present for all participants (Fig. 2). This was true even for the four participants with considerably increased tone, no visible active movement, and minimal passive movement (i.e., MAS score of 3).

**Fig. 3:**
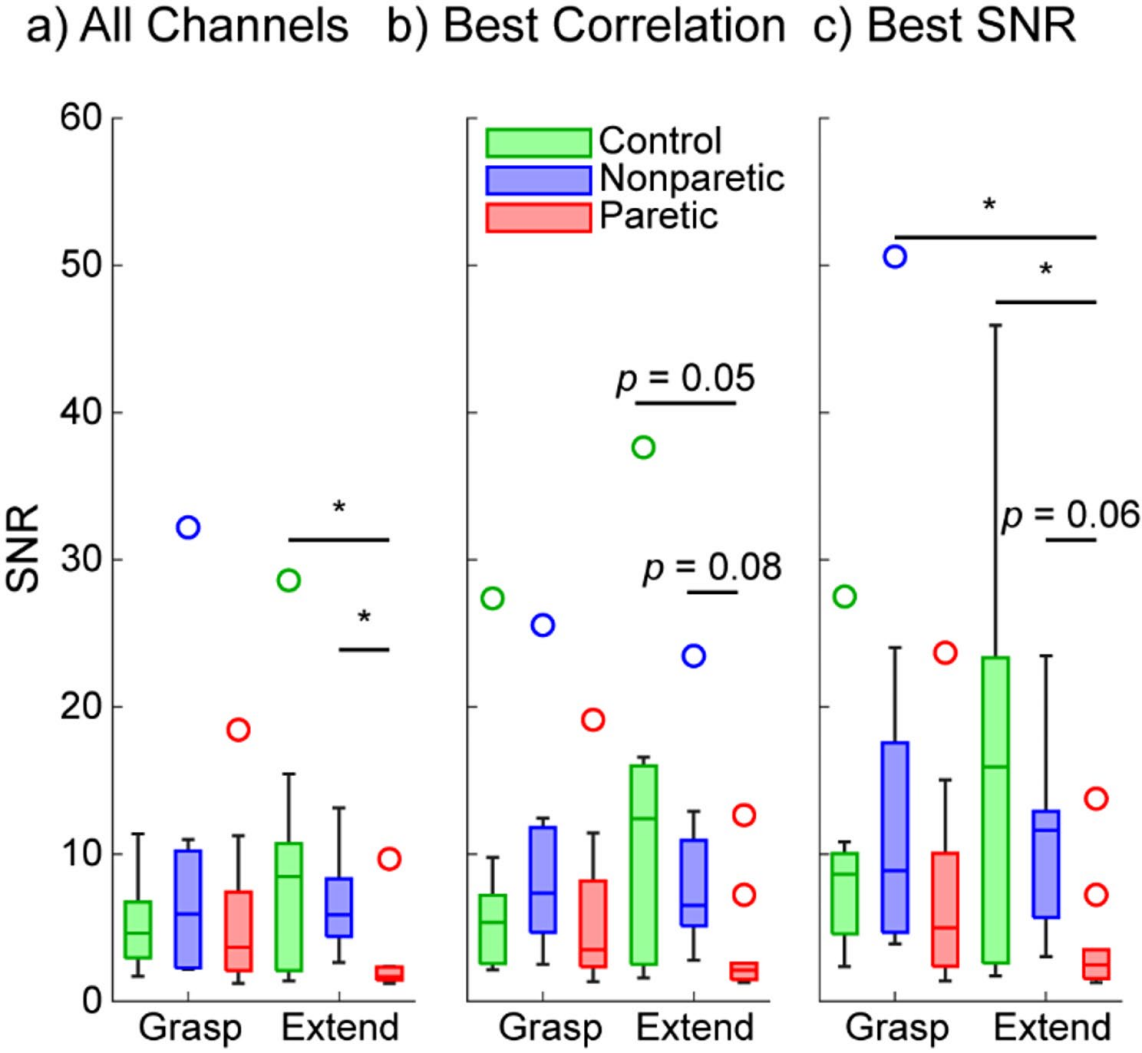
EMG SNR during hand grasp and hand extension using all 32 EMG channels (**a**), the top five most correlated EMG channels (**b**), and top five EMG channels with the highest SNR (**c**). (**a**) Across all 32 EMG channels, the SNR of the paretic EMG was significantly lower than the SNR of the nonparetic EMG and healthy EMG for hand extension. In contrast, no differences were observed among groups for hand grasp. Similarly, no within-group differences were found between hand grasp and hand extension. (**b**) When using only the top five most correlated EMG channels as a proxy for recording directly from the respective flexors or extensors, a similar trend is seen, although the difference in paretic extension is no longer significant. (**c**) When using only the top five EMG channels with the highest SNR as another proxy for recording directly from the respective flexors or extensors, the trend is still present, and extension is significantly worse for the paretic hand than the healthy control group. Box plots show the median, interquartile range, and most extreme non-outlier values. Circles denote outliers. Asterisk (*) denotes *p* < 0.05, pairwise rank-sum tests with correction for multiple comparisons. *N* = 10 control participants and 10 stroke participants (nonparetic and paretic)

We found that in grasp movements, the SNR was not significantly different between the groups (control vs. nonparetic *p* = 0.9926, control vs. paretic *p* = 0.9994, nonparetic vs. paretic *p* = 0.941; pairwise rank-sum tests with correction for multiple comparisons; Fig. 3A). However, in extension movements, the paretic SNR was significantly lower than both nonparetic and control SNR (*p*’s < 0.05, pairwise rank-sum tests with correction for multiple comparisons).

Importantly, these differences in SNR were observed with an ensemble of 32 EMG channels where some channels are not placed directly over a flexor or extensor. To explore the impact of electrode placement on SNR, we next looked at the SNR from only the top five EMG channels correlated to either flexion or extension, as these channels would most likely be placed over a flexor or extensor, respectively. Using this approach, we found a similar trend (Fig. 3B), although no significant differences were present among any of the groups (*p*’s > 0.05, pairwise rank-sum tests with correction for multiple comparisons).

Another approach to explore the impact of electrode placement on SNR is to simply look at the subset of channels with the best SNR for flexion or extension, as these would also likely be placed over a flexor or extensor respectively. To this end, we also looked at the SNR from the top five EMG channels with the best SNR for flexion and extension. A similar trend was found (Fig. 3C), and paretic extension SNR was lower than control extension (*p* = 0.0475) but not lower than nonparetic extension (*p* = 0.0676, pairwise rank-sum tests with correction for multiple comparisons).

We also separated the paretic SNR by whether the participants received botulinum toxin injections in their affected arms. Although our sample size was limited, we found no significant difference between the calculated SNR between the two groups for grasping movements (Supplemental Fig. S1; *p* = 0.48 Wilcoxon rank sum test) or extension movements (Supplemental Fig. S1; *p* = 0.76 Wilcoxon rank sum test).

### High-density EMG and modified Kalman filter improves SNR

As a first-order approach to extracting control from paretic EMG, we quantified the SNR of the output produced by the MKF. The MKF resulted in significantly greater SNR for grasping and extension in both the control and non-paretic groups (*p*’s < 0.05, pairwise rank-sum tests with correction for multiple comparisons). A similar trend was present for the paretic group, although this was not significant (Supplemental Fig. S2).

**Fig. 4:**
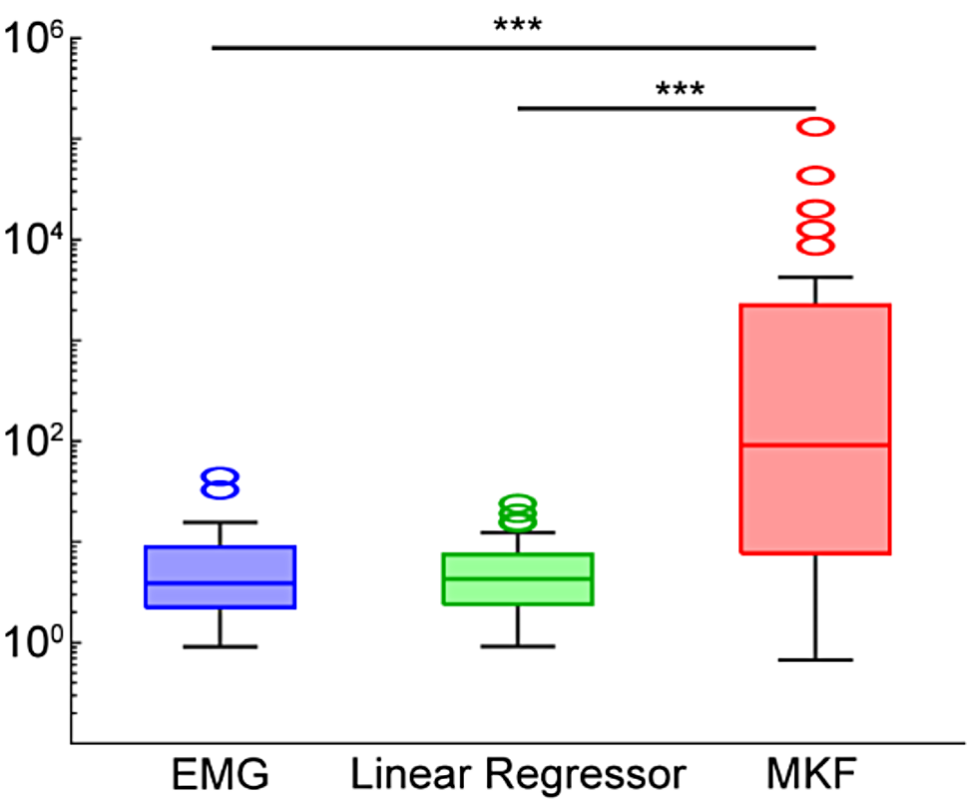
SNR of EMG, linear regression, and MKF output. The MKF had significantly higher SNR than EMG alone and the linear regressor. Data are aggregated across all patient groups (control, nonparetic, and paretic) as well as both motions (flexion and extension). Data are shown on a logarithmic y-axis. Box plots show the median, interquartile range, and most extreme non-outlier values. Circles denote outliers. Triple asterisk (***) denotes *p* < 0.001, pairwise rank-sum tests with correction for multiple comparisons. *N* = 10 control participants and 10 stroke participants (nonparetic and paretic)

Using aggregate data from all patient groups and both flexion and extension motions, the MKF resulted in significantly higher SNR than the EMG signal alone (*p* < 0.001, pairwise rank-sum tests with correction for multiple comparisons; Fig. 4). The SNR of the MKF was also significantly higher than the SNR of a simple linear regressor using the most correlated EMG channel (*p* < 0.001, pairwise rank-sum tests with correction for multiple comparisons; Fig. 4). Thus, simple linear regression from one EMG channel placed directly over the flexors or extensors, as is typical for commercial myoelectric prostheses, may not be sufficient for proportional control alone.

### Stroke survivors retain the ability to finely regulate muscle activity even in extreme cases of spasticity where the hand is completely immobile

**Fig. 5:**
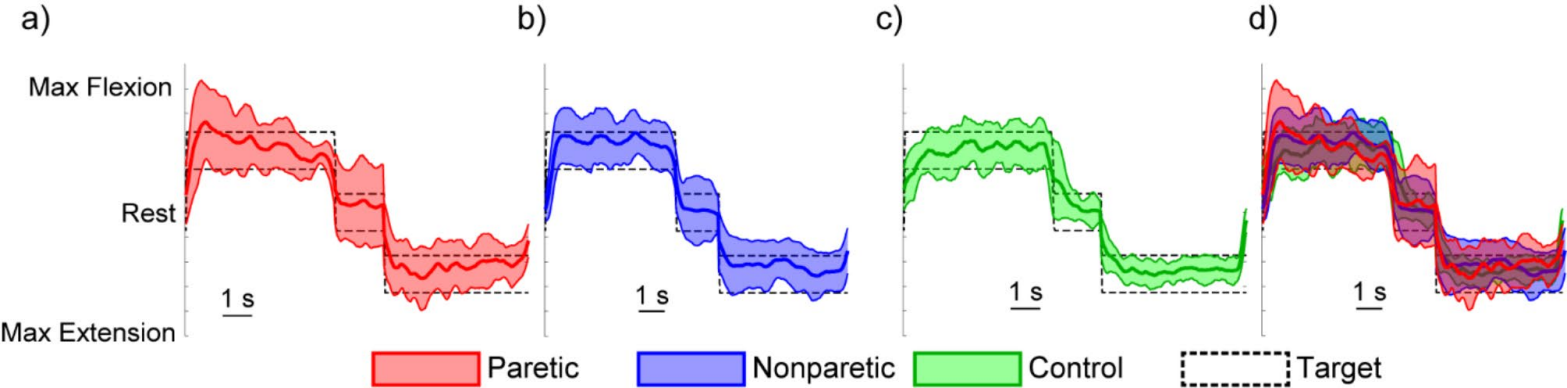
Performance of the virtual target-touching task for one of the stroke participant’s paretic arm (**a**; red) and nonparetic arm (**b**; blue). Data from a representative control is shown separately (**c**; green) and overlaid with the stroke participant’s data (d) for comparison. The task was achievable under all conditions, as indicated by the bold lines staying within the target window. Data show the kinematic position of a virtual bionic hand while attempting to perform a partial hand grasp (50% output), followed by a brief period of rest, and then a partial hand extension (50% output). The dotted lines represent the target window the participants attempted to remain within. Data show the mean and standard deviation of the kinematic position across the 20 trials of the task. The healthy participant and stroke participant shown were at the mean performance level of their respective groups. Notably, the stroke participant at the mean performance level, for which the data is shown, had an MAS score of 3, indicating minimal hand motion

Next, we tasked stroke survivors with spastic hemiparesis to complete a proportional target-touching task using EMG-based control. All 10 stroke survivors successfully completed the task, demonstrating their ability to finely regulate their muscle activity to stay within the error window (Fig. 5). This was true for both hand grasp and for hand extension, despite there being significantly worse EMG SNR for extension on the paretic side (Fig. 3). Furthermore, the four participants with considerably increased tone, no visible active movement, and minimal passive movement (i.e., MAS score of 3) were also able to successfully achieve proportional EMG control. Indeed, Fig. 5 shows representative performance on the task from a stroke survivor at the mean performance level, who coincidentally had an MAS score of 3.

### Proportional EMG control from paretic arms is similar to nonparetic and healthy arms for grasping

**Fig. 6:**
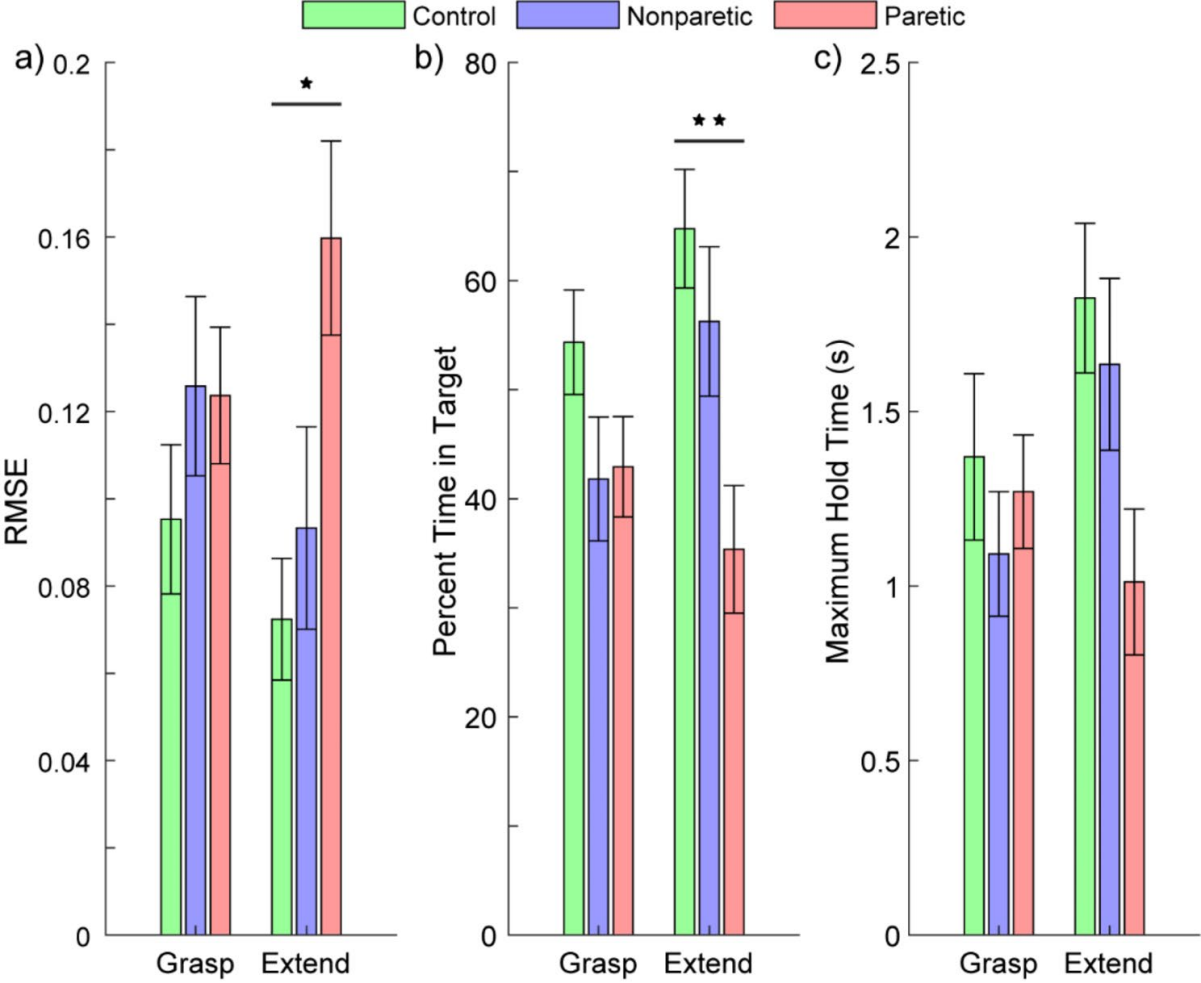
Proportional EMG control of hand grasp and extension for paretic, nonparetic, and healthy arm. Participants completed a target-touching task with a virtual bionic arm controlled by surface EMG from the extrinsic hand muscles. Performance on the task was measured using the RMSE between the participant’s kinematic position and the target position (**a**), the percent time within the target window (**b**), and the maximum continuous duration within the target window (**c**). Lower RMSE indicates better performance. A higher percent time within the target window and a longer maximum hold time indicate better performance. Across all three metrics, no significant differences were observed for grasping among the paretic, nonparetic, and healthy arms. In contrast, the paretic arm had significantly worse RMSE and percent time within the target window for hand extension. Asterisk (*) denotes *p* < 0.05, double asterisk (**) denotes *p* < 0.01, pairwise comparisons with correction for multiple comparisons. *N* = 10 healthy controls and 10 stroke participants (nonparetic and paretic). Data show mean ± standard error of the mean

Having shown that stroke survivors could reliably achieve proportional EMG control with their paretic arm, we next quantified task performance relative to their nonparetic arm and healthy controls. We found no significant difference in task performance between paretic, nonparetic, and healthy arms when performing hand grasp (Fig. 6). This was true for all three performance metrics; there was no significant difference among RMSE (*p* = 0.4157, ANOVA), the percent time within the target (*p* = 0.1713, ANOVA), or the maximum hold time (*p* = 0.6009, ANOVA).

In contrast, for proportional hand extension, we observed significantly worse performance with the paretic arm compared to healthy controls. This was true for both RMSE (*p* < 0.05) and the percent time within the target (*p* < 0.01, pairwise unpaired *t*-tests with correction for multiple comparisons). No significant difference was observed between the paretic and nonparetic arm, or between any of the groups for the maximum hold time.

We also quantified each participant’s variance among their attempts at the task (Supplemental Fig. S3). A smaller variance would indicate more precise and consistent control. We found no significant differences among the patient populations; paretic, non-paretic, and healthy hands had similar precision at the task. However, we did see a significant difference between grasping and extension for RMSE and percent time in target at the aggregate level (*p* < 0.05 and *p* < 0.01 respectively, pairwise comparisons with correction for multiple comparisons). That is, all participants had less precision with grasping than with extension. For the paretic hand of stroke patients, this implies extension has worse accuracy but grasping has worse precision.

Importantly, despite the heterogeneity of stroke, we found no significant difference among the variance of the patient groups. In other words, the relative performance among stroke patients with their paretic limb was no different than the relative performance among stroke patients with their nonparetic limb or among healthy controls (*p*’s > 0.15 for all metrics, F-tests with correction for multiple comparisons).

Post-hoc analyses regarding the use of botulinum toxin injections also suggest no significant impact on proportional EMG control. No significant differences were observed in any of the metrics for both grasping and extension (*p*’s > 0.3, unpaired *t*-tests; Supplemental Fig. S4).

### Proportional EMG control is not correlated with spasticity, age, or time since stroke

**Fig. 7:**
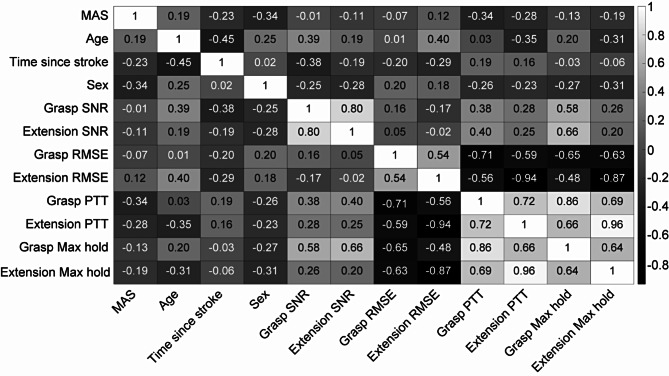
Paretic EMG SNR and task performance were not correlated with spasticity, age, or time since stroke. The heatmap shows pairwise Pearson correlation coefficients

We also calculated the Pearson correlation coefficients between paretic EMG SNR, task performance metrics, and participant demographics (Fig. 7). We found no meaningful correlation among participant demographics, suggesting that, at least for these participants, proportional EMG control was not dependent on spasticity, age, or time since the stroke. Some moderate correlations (absolute values of 0.4 to 0.6) were observed among EMG SNR and task performance metrics. Some strong correlations (absolute values of 0.6 to 0.8) were observed between grasping and extension metrics. A few extremely strong correlations (absolute values of 0.8 to 1) were observed among performance metrics for a given condition (e.g., grasping RMSE vs. grasping PTT).

Given that some effects, such as age and sex would likely impact both the paretic and nonparetic arm equally, we furthered this analysis by calculating the Pearson correlation coefficients between nonparetic EMG SNR, task performance metrics, and participant demographics (Supplemental Fig. S5). We found similar trends between the paretic and non-paretic side. However, on the paretic side SNR was correlated to task performance, and this was not true for the non-paretic side.

## Discussion

Prior work has shown it is possible to classify multiple discrete hand gestures from paretic EMG after a stroke [29, 60]. In contrast, here we show that it is also possible to proportionally regress kinematic position from paretic EMG after a stroke. Importantly, all participants, regardless of the severity of their post-stroke spasticity, were able to achieve proportional position control. This was true even for patients with MAS scores of 3 who were unable to physically move their hand. Together, these findings suggest that it is feasible to provide more dexterous EMG control of assistive devices for stroke patients, as proportional control could also be extended to velocity, force, or torque control. These findings are particularly impactful and timely given the increasing prevalence of stroke [75], the growing popularity of powered orthotics [76, 77], and the new reimbursement pathway for powered upper-limb orthotics [78].

Prior work has shown that impaired movement is correlated with higher spasticity and MAS scores [79]. In contrast, here, we show that proportional EMG control is not correlated with MAS scores. In other words, patients can still selectively modulate EMG activity even in severe cases of spasticity where there is no overt hand movement. These patients with severe spasticity are typically ineligible for assistive powered hand orthoses due to the excessive torque necessary to overcome their spasticity [80]. However, the use of assistive EMG-controlled exoskeletons has been shown to improve arm function [40, 81, 82]. Thus, an unfortunate reality is that the patients who could benefit the most from an exoskeleton are paradoxically unqualified to receive them. The fact that proportional EMG control is possible with extreme spasticity supports the use of EMG-based virtual reality [83] and/or biofeedback [84, 85] therapy to improve arm function, thereby helping patients qualify for assistive powered hand exoskeletons.

The use of novel EMG therapies for spasticity is further supported by the fact that EMG control was not hindered by routine clinical management of spasticity via botulinum toxin injections. This finding is consistent with prior showing that botulinum toxin injections improve voluntary motor control [86], have no impact on the motor performance of the spastic muscles [87], and can improve EMG pattern-recognition control [88]. Botulinum toxin has also been shown to reduce muscle activity overall [86, 87, 89, 90], but muscle activity recovers within a few weeks [89]. This finding, however, is limited by our small sample size; lack of a statistical difference does not imply equivalence. Future work should investigate the impact of botulinum toxin on EMG control in a larger cohort of patients within the first few hours and days after injection.

Although we show that proportional EMG control is possible for both hand grasp and hand extension, we also show that proportional EMG control for the paretic hand was more accurate, but less precise for grasping relative to extension. These results are consistent with the underlying neurophysiology of post-stroke spasticity. After a stroke, there is an increase in inappropriate muscle coactivation [61], and hand extension is often more impacted than hand grasp [61, 91]. Future implementations of proportional EMG control for assistive devices should leverage this knowledge to design EMG control algorithms around hand grasp instead of hand extension, akin to a voluntary-close prosthesis [92] or orthosis [40, 93, 94].

In this study, we used high-density EMG and a modified Kalman filter to extract proportional position control from paretic EMG. An important question is whether or not proportional position control could be achieved using fewer EMG channels. Preliminary analyses suggest that, at least for hand grasping and extension, fewer channels may actually be preferential for the paretic arm (Supplemental Fig. S6). Indeed, we observed that the RMSE of the MKF initially decreased and then plateaued as more channels were added for the healthy and non-paretic arms. In contrast, performance degraded as the number of channels increased for the paretic arm. Although it is uncertain how these offline measures of RMSE will translate to real-world human-in-the-loop control, future work should explore the optimal number and placement of EMG channels.

This study focused exclusively on hand grasping and hand extension as these motions are fundamental to activities of daily living, enable grip force regulation, and are readily supported by existing assistive hand orthoses. Future work should explore the ability to provide proportional position control over multiple motions simultaneously. The MKF used in this study has been used to provide simultaneous and proportional control of six degrees of freedom of the hand in real-time for healthy and amputee populations [46]. RMSE of the MKF increases as the number of degrees of freedom increases [95]. In our preliminary analyses, we found the rate of decline may be accelerated for the paretic arm (Supplemental Fig. S7). Future work should consider more advanced non-linear control algorithms, as these have already shown the capacity to classify multiple hand gestures with high accuracy from the paretic EMG of stroke survivors [29, 60].

## Conclusion

Here, we show that stroke survivors can achieve proportional EMG control, regardless of their age, time since their stroke, clinical spasticity rate, and history of botulinum toxin injections. We also show that EMG signal-to-noise ratio and proportional control are better for grasping motions than extension motions. This work constitutes an important step towards the advancement of more intuitive and dexterous hand exoskeletons with proportional position control. More dexterous upper-limb assistive powered orthoses may ultimately improve the quality of life of stroke survivors.

## Electronic supplementary material

Below is the link to the electronic supplementary material.

Supplementary Material 1

## Data Availability

The data supporting this study’s findings are available upon reasonable request to the authors.

## Abbreviations

EMG: Electromyography
SNR: Signal-to-noise ratio
MAS: Modified Ashworth Scale
MKF: Modified Kalman Filter
RMSE: Root-mean-square error
TTT: Target-touching task
