# Supplementary material for: Proportional myoelectric control of a virtual bionic arm in participants with hemiparesis, muscle spasticity, and impaired range of motion
Source: J Neuroeng Rehabil. 2024 Dec 21;21:222. doi: 10.1186/s12984-024-01529-0 (PMC11662728; doi:10.1186/s12984-024-01529-0)
Supplement: Supplementary file 1 — Supplementary Material 1 [file 12984_2024_1529_MOESM1_ESM.docx]

**Supplemental Materials**


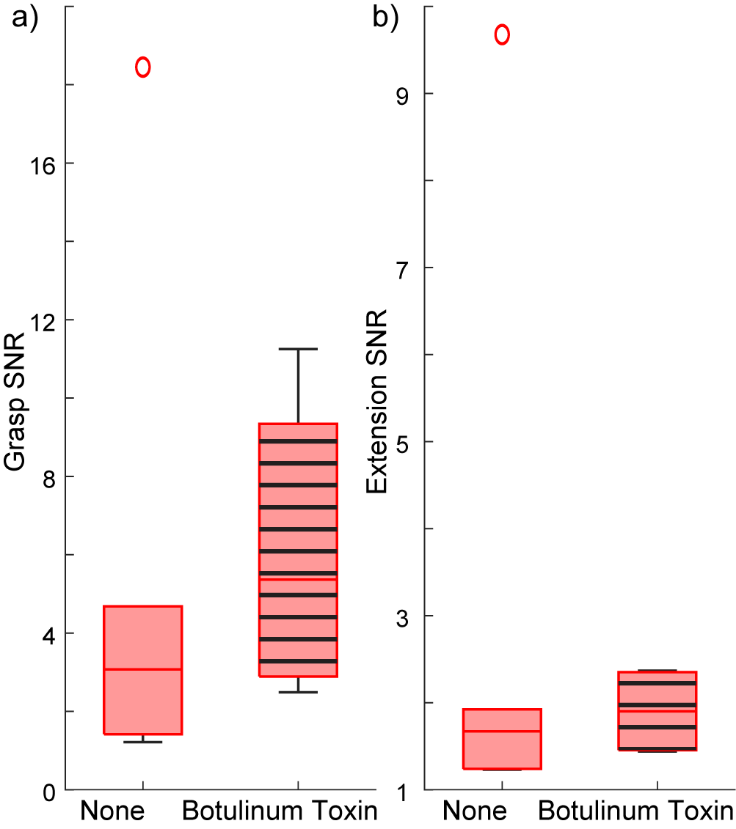


Supplemental Figure S1. Botulinum toxin injections had no significant effect on EMG SNR in either grasping (a) or extension (b). Box plots show the median, interquartile range, and most extreme non-outlier values. Circles denote outliers. N = 6 participants without injections and N = 4 participants with botulinum toxin injections. Note lack of significance does not imply equivalence; data are limited by the small sample size.


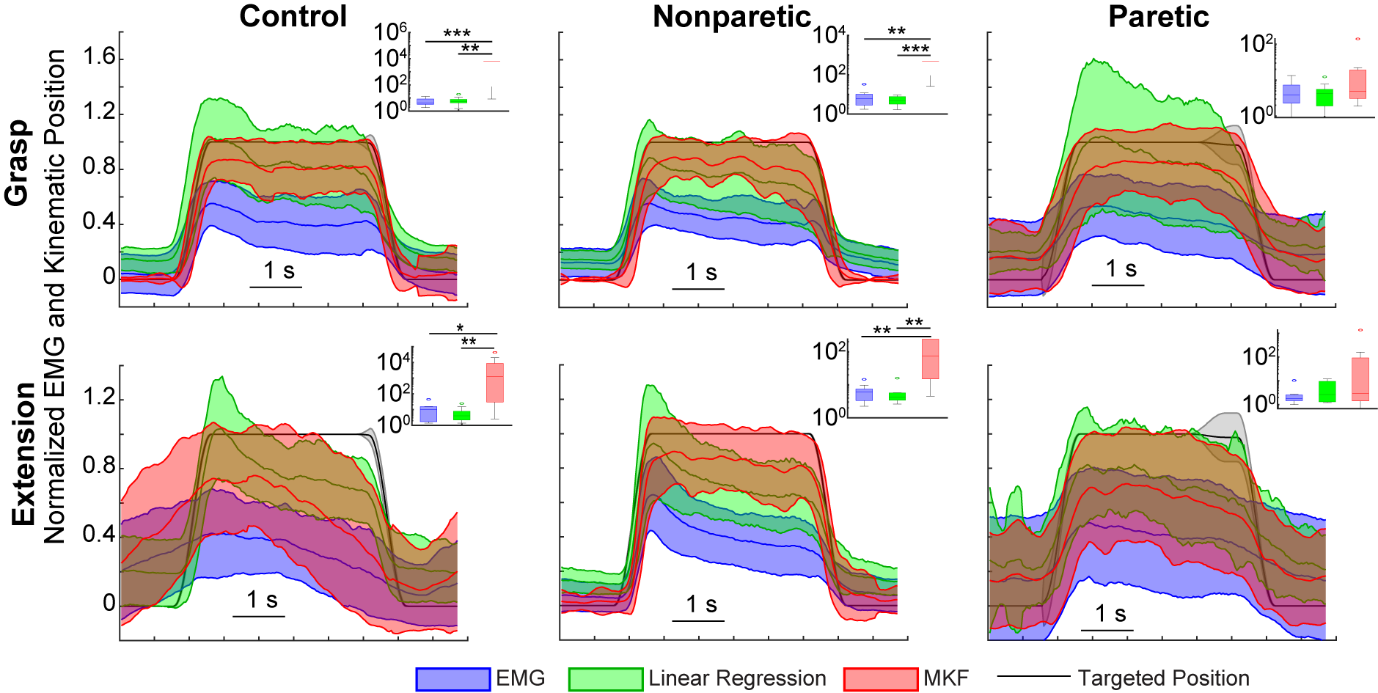


Supplemental Figure S2.Normalized EMG activity of extrinsic hand muscles (blue) during instructed hand grasp (top row) and extension (bottom row) and predicted hand position for linear regression based on the most correlated EMG channel (green) and for a modified Kalman filter (MKF; red). Insets show the signal-to-noise ratio for the EMG and for the predictions. The SNR of the predicted positions from the MKF were often significantly higher than linear regression and the EMG. Box plots show the median, interquartile range, and most extreme non-outlier values. Circles denote outliers. Asterisk (*) denotes *p* < 0.05, double asterisk (**) denotes *p* < 0.01, and triple asterisk (***) denotes *p* < 0.001, pairwise rank-sum tests with correction for multiple comparisons. N=10 control participants and 10 stroke participants (nonparetic and paretic).


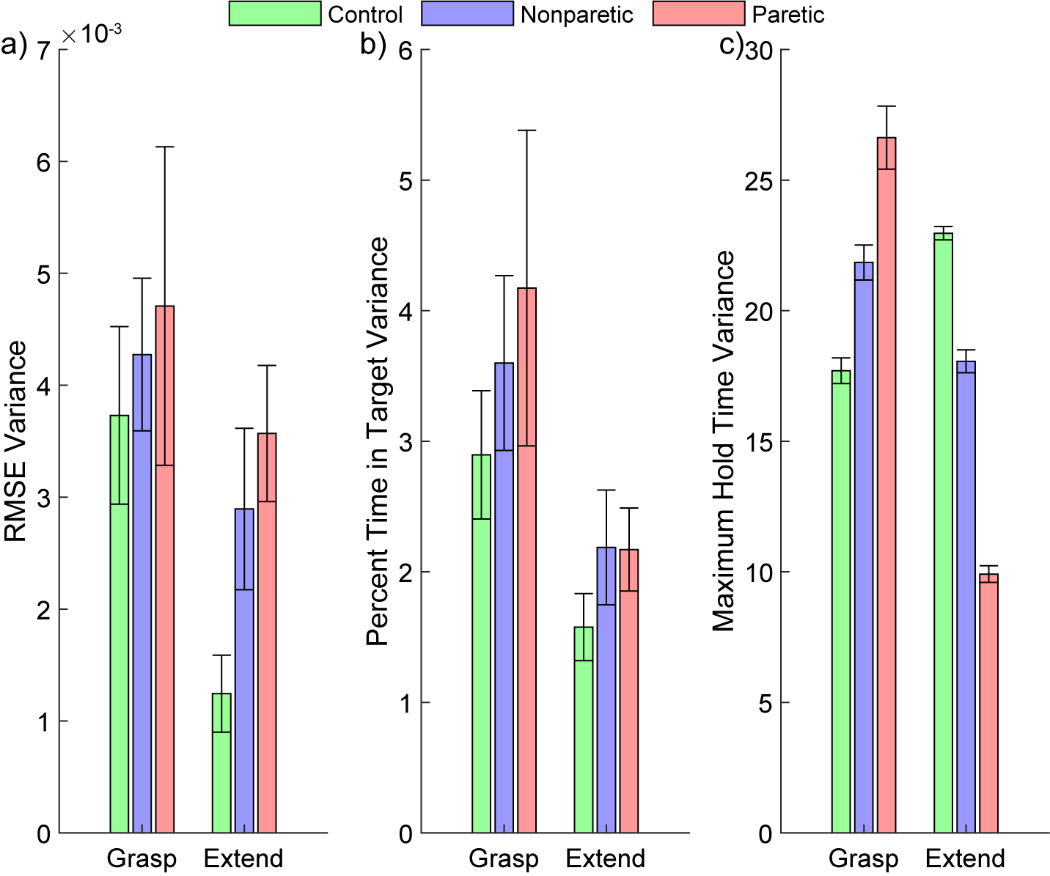


Supplemental Figure S3. Variance among attempts at the task for each participant. No significant differences were found among participant groups. In RMSE and percent time in target, aggregate data from all participant groups show a significant difference between grasping and extension (*p* < 0.05 and *p* < 0.01 respectively; pairwise comparisons with correction for multiple comparisons). No other significant differences were found. Data show mean ± standard error of the mean. N=10 control participants and 10 stroke participants (nonparetic and paretic).


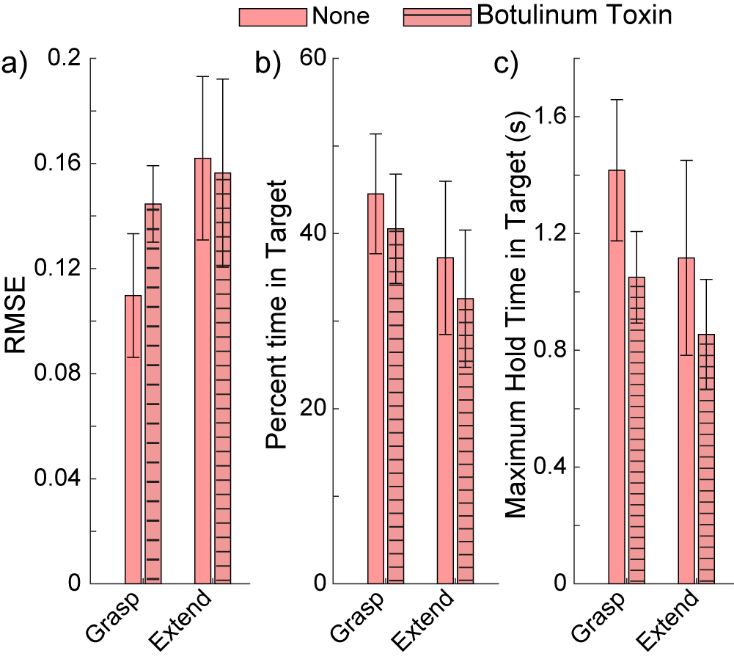


Supplemental Figure S4. Botulinum toxin injections had no significant impact on proportional EMG control. Lower RMSE indicates better performance (a). A higher percent time within the target window (b) and a longer maximum hold time indicate better performance (c). Data show mean ± standard error of the mean. N = 6 participants without injections and N = 4 participants with botulinum toxin injections. Note lack of significance does not imply equivalence; data are limited by the small sample size.


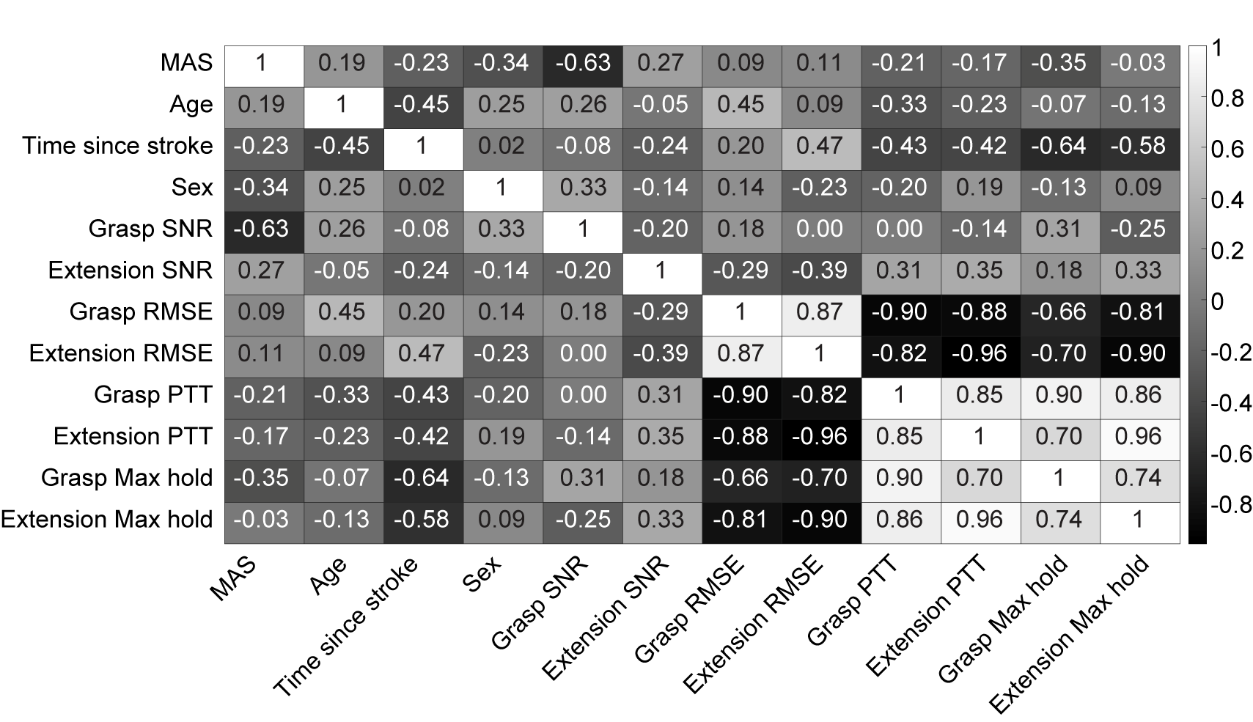


Supplemental Figure S5. Nonparetic EMG SNR and task performance were generally not correlated with the spasticity of the contralateral limb, age, or time since stroke. The heatmap shows pairwise Pearson correlation coefficients.


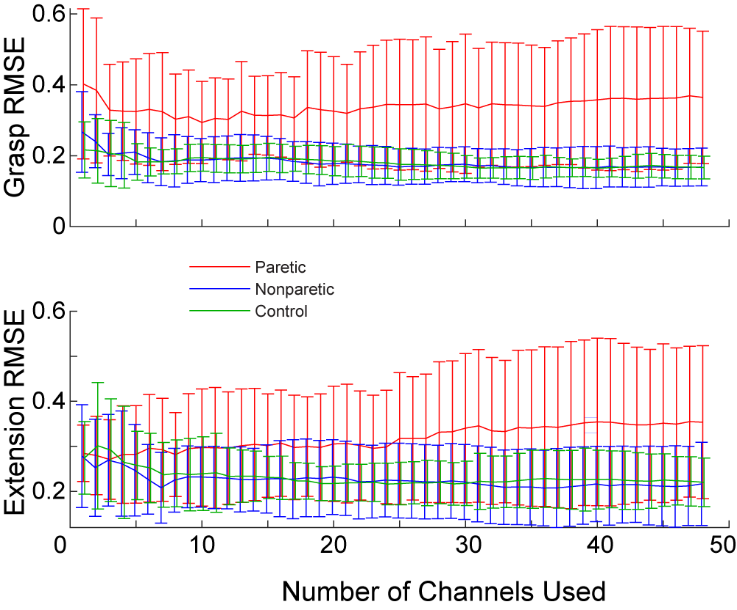


Supplemental Figure S6. RMSE of the MKF as a function of number of EMG channels used. Performance increases as the number of channels increases, up to ~7 channels. Beyond 7 channels, performance plateaus for the healthy and non-paretic conditions, and degrades for the paretic condition. Channels were selected using a Gram-Schmitt orthonormalization algorithm. Lines show the mean RMSE and error bars show the standard deviation. N=10 control participants and 10 stroke participants (nonparetic and paretic).


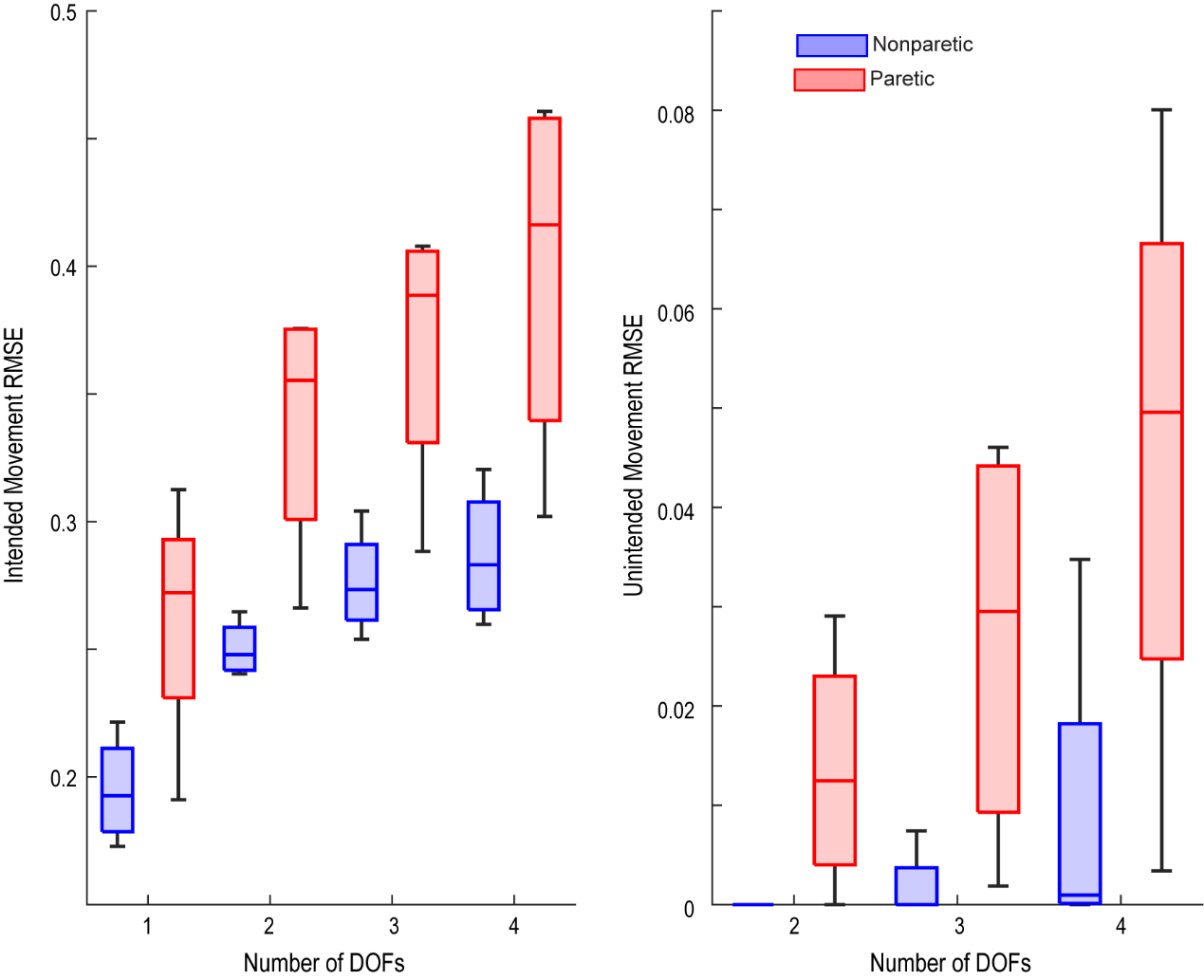


Supplemental Figure S7. RMSE of the MKF as a function of controllable degrees of freedom (DOFs). Preliminary offline analyses from four stroke patients show that the RMSE increases as the number of controllable degrees of freedom increases, and that the rate of increase may be accelerated on the paretic side. No significant differences found between groups, although this is likely attributed to the lack of statistical power due to the small sample size. Degrees of freedom include: D1-D3 flexion/extension (tripod pinch), D4-D5 flexion/extension, wrist flexion/extension, and wrist pronation/supination. Intended movement RMSE quantifies the RMSE of the select DOF during isolated movement, and unintended movement RMSE quantifies the RMSE of all other DOFs during the movement of the select DOF. Box plots show the median, interquartile range, and most extreme non-outlier values N=4 stroke participants.
